# Renal autotransplantation to treat renal artery aneurysm: case report

**DOI:** 10.1590/1516-3180.2014.1325678

**Published:** 2014-07-29

**Authors:** Tercio Genzini, Huda Maria Noujaim, Leonardo Toledo Mota, Luiz Estevam Ianhez, Rodrigo Azevedo de Oliveira, Erica Takako Muramoto Shiroma, Fernando Towata, Marcelo Perosa de Miranda

**Affiliations:** I MD, MSc. Director of Hepato Group, Hospital Bandeirantes, São Paulo, Brazil; II MD, PhD. Attending physician, Hepato Group, Hospital Bandeirantes, São Paulo, Brazil; III MD. Attending physician, Hepato Group, Hospital Bandeirantes, São Paulo Brazil; IV MD. Attending physician, Hepato Group, Hospital Bandeirantes, São Paulo, São Paulo, Brazil; V Medical Student, Faculdade de Medicina do ABC (FMABC), São Paulo, Brazil

**Keywords:** Aneurysm, Renal artery, Transplantation, General surgery, Kidney, Aneurisma, Artéria renal, Transplante, Cirurgia geral, Rim

## Abstract

**CONTEXT:**

Renal artery aneurysm (RAA) is uncommon and usually asymptomatic, but complications like rupture or thromboembolism of the aneurysm can occur, with consequent renal infarction. Most of the clinical findings are found incidentally through imaging examinations, in investigating other diseases. Renal autotransplantation (RAT) is an alternative treatment for complex RAA, with satisfactory results described in the literature.

**CASE REPORT:**

The patient was a 48-year-old man with a history of systemic arterial hypertension, thrombocytopenia and advanced hepatosplenic schistosomiasis. He complained of right lumbar pain, which was investigated through imaging examinations (computed tomography and angiotomography). These revealed right RAA of 2.5 cm in diameter. Evaluation by the vascular surgery team found that this was untreatable using endovascular methods. The treatment performed was open right nephrectomy with kidney preservation in solution, followed by aneurysmectomy, suturing of the injured artery and kidney reimplantation in the right iliac fossa with anastomosis of the iliac vessels and ureter. The durations of the surgery and kidney ischemia were 385 and 140 minutes, respectively. The patient was discharged on the 20^th^ postoperative day, with creatinine concentration of 1.4 mg/dL, urea 41 mg/dL, urine volume 1400 mL/24 h and ascites treated with diuretics.

**CONCLUSION:**

RAT is indicated basically in three situations: extracorporeal reconstruction of complex aneurysms of the renal pedicle, extensive ureteral injury, and conservative kidney cancer surgery in patients with a single kidney. This study presents a case of a patient with advanced liver disease and RAA that was untreatable using endovascular methods and was successfully treated using RAT.

## Abstract (pt)

**CONTEXTO:**

O aneurisma de artéria renal (AAR) é incomum e, em geral, assintomático, mas podem ocorrer complicações como rotura ou embolia de trombos do aneurisma com consequente infarto renal. A maioria dos achados clínicos é encontrada acidentalmente por exames de imagem na investigação de outras doenças. O autotransplante renal (ATR) constitui-se em alternativa de tratamento de AAR com resultados satisfatórios descritos na literatura.

**RELATO DE CASO:**

Paciente masculino, 48 anos, com histórico de hipertensão arterial sistêmica, plaquetopenia e esquistossomose hepatoesplênica avançada. Referia dor lombar direita que após exames de imagem (tomografia computadorizada e angiotomografia) revelou AAR direita com 2,5 cm de diâmetro não tratável por via endovascular após avaliação da equipe de cirurgia vascular. O tratamento realizado foi uma nefrectomia aberta direita com preservação renal em solução, seguida de aneurismectomia, sutura da artéria lesada e reimplante do rim na fossa ilíaca direita com anastomoses dos vasos ilíacos e do ureter. O tempo cirúrgico e de isquemia renal foram de 385 e 140 minutos, respectivamente. Recebeu alta hospitalar no vigésimo dia do pós-operatório, com concentrações de creatinina de 1,4 mg/dL, ureia de 41 mg/dL, volume urinário de 1400 mL/24 h e ascite tratada com diuréticos.

**CONCLUSÃO:**

O ATR está indicado basicamente em três casos: reconstrução extracorpórea de aneurismas complexos do pedículo renal, lesão ureteral extensa e cirurgia conservadora de câncer renal em pacientes com único rim. Este estudo apresenta caso de paciente com doença hepática avançada e AAR intratável por método endovascular e tratado com sucesso por ATR.

## INTRODUCTION

Renal artery aneurysm (RAA) is unusual and occurs in approximately 0.09% of the population.^1^ In general, this condition is asymptomatic, but severe complications such as rupture, embolism or thrombus expansion of the aneurysm with consequent renal infarction may occur. It is often diagnosed accidentally and is done through imaging tests such as computed tomography (CT) and arteriography during investigations of other diseases. Epidemiologically, it more often affects women and the left kidney; it is typically solitary and associated with fibromuscular dysplasia.^2^^,^ 3 The indications for treatment should take into consideration the patient's age, sex, blood pressure and renal function, and the size of the aneurysm. Most often, a size of 2 cm has been considered to be the threshold for endovascular repair.2 Thus, renal autotransplantation (RAT) has emerged as an alternative for treating complex RAA.^4^^,^ 5

## CASE REPORT

**Figure 1:**
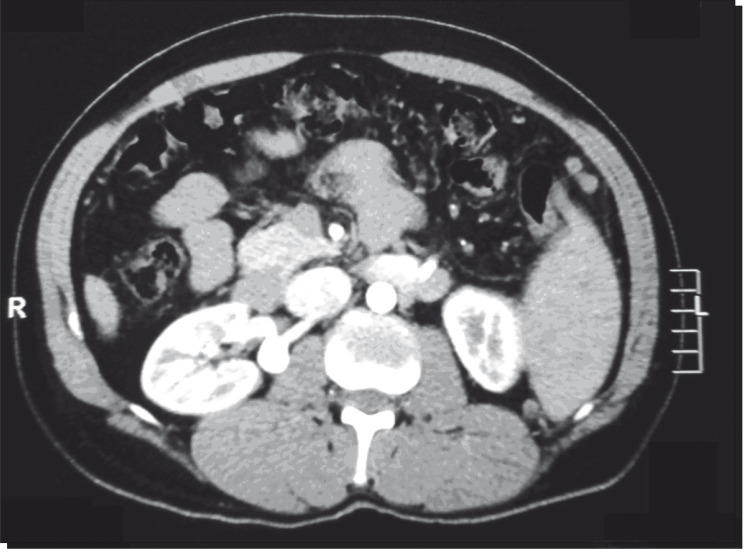
Computed tomography showing right renal artery aneurysm.

**Figure 2:**
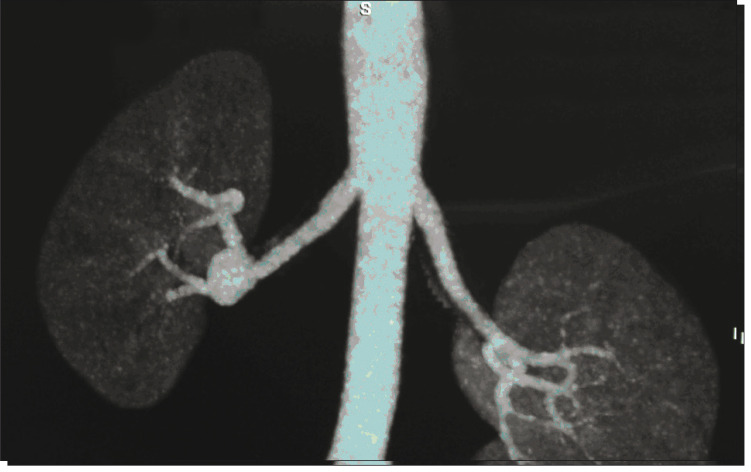
Computed tomography arteriography showing aneurysm of approximately 2.5 cm in the right renal artery.

The patient was a 48-year-old man with a previous history of high blood pressure and thrombocytopenia and a diagnosis of advanced hepatosplenic schistosomiasis. He reported having right lumbar pain, and this was found to be caused by an RAA of 2.5 cm in diameter, which was observed on CT and CT arteriography (Figures 1 and 2). The preoperative serum creatinine level was 1.8 mg/dl.

**Figure 3:**
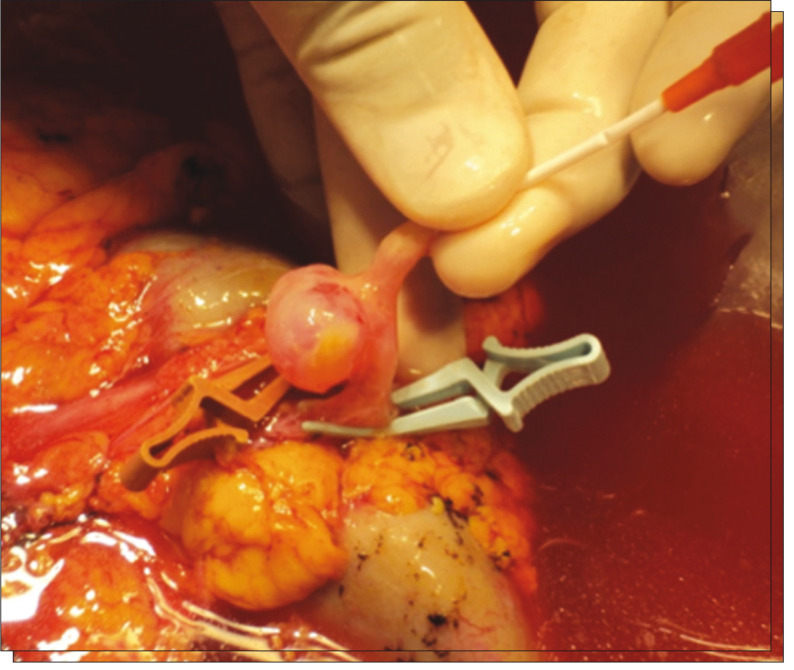
Renal artery aneurysm of 2.5 cm in diameter.

**Figure 4:**
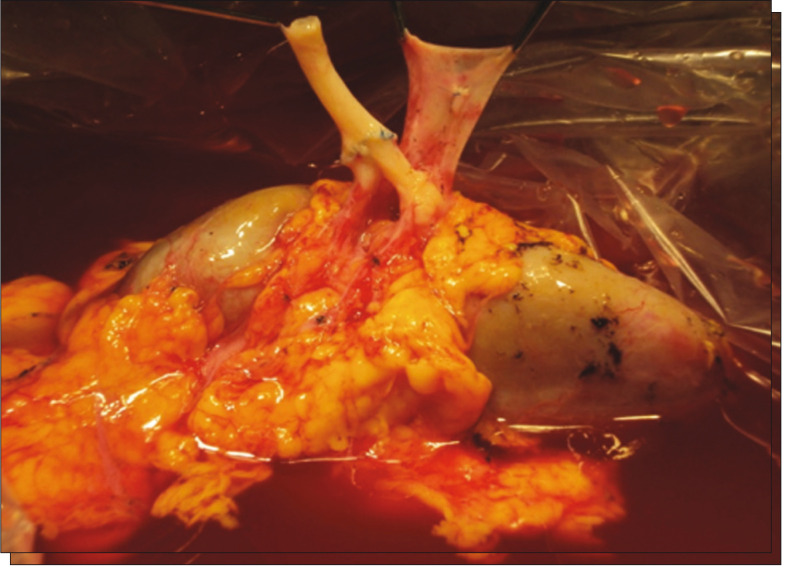
End-to-end anastomosis for reconstruction of the renal artery.

The patient was referred to the department of vascular surgery, but the RAA was considered to be untreatable using an endovascular approach. RAT was performed by the transplantation group. The treatment consisted of right nephrectomy and preservation of the kidney in Euro-Collins solution, followed by aneurysmectomy and artery reconstruction by means of end-toend anastomosis between the renal artery and the hilar branches using 7/0 Prolene suturing on the back table (Figures 3 and 4).

Kidney reimplantation was performed in the right iliac fossa. A renal vein graft was anastomosed end-to-side with the right external iliac vein using 5/0 Prolene suturing and the reconstructed renal artery graft was anastomosed end-to-end with the hypogastric artery using 7/0 Prolene suturing. Ureter anastomosis was performed using the modified Lich Gregoir technique on the anterolateral side of the bladder. The operation lasted 385 minutes and the time of ischemia renal graft was 140 minutes. The patient was extubated in the operating room and transferred to the intensive care unit, where he stayed for six days due to ascites caused by liver disease decompensation. He was discharged on the 20^th^ postoperative day, with a creatinine level of 1.4 mg/dl, urea of 41 mg/dl, urine volume 1400 ml/24 h and ascites treated with diuretics. Currently, the patient is symptom-free, as seen at the 34^th^ month follow-up with normal renal function (creatinine = 1.0 mg/dl).

## DISCUSSION

In most cases, renal artery aneurysm does not cause any clinical symptoms, but some nonspecific signs such as lumbar pain may be present. In suspected cases, imaging tests should be performed and digital subtraction angiography is the best diagnostic test.^6^ When an aneurysm is identified, surgery is the best treatment option, in order to avoid hypertension or rupture of the aneurysm, especially in cases in which the aneurysm is larger than 2 cm in diameter and considered to comprise complex RAA.^2^

RAT is mainly indicated in three situations: extracorporeal reconstruction of complex aneurysms of the renal pedicle; extensive ureteral injury; and conservative surgery due to kidney cancer in patients with only one kidney. Furthermore, RAT should be considered for treating renal artery aneurysm that is found to be untreatable by means of endovascular methods. Some reports in the literature have shown that RAT was effective in treating complex renal artery aneurysm^7^^,^ 8 (Table 1). 

**Table 1:** Search strategies performed in February 2012 and results from Pubmed, Embase, Lilacs (Literatura Latino Americana e do Caribe em Ciências da Saúde) and the Cochrane Library regarding the topic of renal autotransplantation in cases of renal aneurysms

Database	Search terms	Results	Relevant findings
MedLine (via Pubmed)	(Renal) AND (transplantation) AND (aneurysm) with case report filter	382	Use of *ex vivo* repair or vein graft for arterial reconstruction of the aneurysmectomy
			Laparoscopic nephrectomy with backbench *ex vivo* repair followed by autotransplantation through a small laparoscopic extraction incision
Lilacs (via Bireme)	(Renal) AND (transplantation) AND (aneurysm)	40	Renal autotransplantation is indicated in cases of complex aneurysms or ureteral injury
	(Rim) AND (transplante) AND (aneurisma)	11	Vascular reconstruction bypass is indicated when the artery aneurysms cannot be corrected by endovascular treatment or *in situ*
Embase (via Elsevier)	(Renal transplantation) AND (aneurysm)	250	Most symptom-free aneurysms < 2.5 cm in diameter can be safely treated expectantly

Recently, the laparoscopic approach to nephrectomy has been increasingly used.^9^ In our case, we chose to use a surgical and non-endovascular approach towards treating RAA, because in our case it was too close to the bifurcation of the hilar renal artery and, especially, because of the need to preserve the kidney, given that schistosomiasis liver disease and portal hypertension can impair renal function. Anticoagulation was not used because there was thrombocytopenia and liver failure consequent to schistosomiasis. 

## CONCLUSION

In patients with a clinical risk of renal disorder following nephrectomy, RAT should be considered for treating RAA that is untreatable using endovascular methods.
